# Joint Assimilation of Leaf Area Index and Soil Moisture from Sentinel-1 and Sentinel-2 Data into the WOFOST Model for Winter Wheat Yield Estimation

**DOI:** 10.3390/s19143161

**Published:** 2019-07-18

**Authors:** Haizhu Pan, Zhongxin Chen, Allard de Wit, Jianqiang Ren

**Affiliations:** 1 Key Laboratory of Agricultural Remote Sensing, Ministry of Agriculture, Institute of Agricultural Resources and Regional Planning, Chinese Academy of Agricultural Science, Beijing 100081, China; 2 Wageningen Environmental Research, P.O. Box 47, 6700 AA Wageningen, The Netherlands

**Keywords:** Sentinel-1 and Sentinel-2, LAI, SM, WOFOST, data assimilation, EnKF, winter wheat yield

## Abstract

It is well known that timely crop growth monitoring and accurate crop yield estimation at a fine scale is of vital importance for agricultural monitoring and crop management. Crop growth models have been widely used for crop growth process description and yield prediction. In particular, the accurate simulation of important state variables, such as leaf area index (LAI) and root zone soil moisture (SM), is of great importance for yield estimation. Data assimilation is a useful tool that combines a crop model and external observations (often derived from remote sensing data) to improve the simulated crop state variables and consequently model outputs like crop total biomass, water use and grain yield. In spite of its effectiveness, applying data assimilation for monitoring crop growth at the regional scale in China remains challenging, due to the lack of high spatiotemporal resolution satellite data that can match the small field sizes which are typical for agriculture in China. With the accessibility of freely available images acquired by Sentinel satellites, it becomes possible to acquire data at high spatiotemporal resolution (10–30 m, 5–6 days), which offers attractive opportunities to characterize crop growth. In this study, we assimilated remotely sensed LAI and SM into the Word Food Studies (WOFOST) model to estimate winter wheat yield using an ensemble Kalman filter (EnKF) algorithm. The LAI was calculated from Sentinel-2 using a lookup table method, and the SM was calculated from Sentinel-1 and Sentinel-2 based on a change detection approach. Through validation with field data, the inverse error was 10% and 35% for LAI and SM, respectively. The open-loop wheat yield estimation, independent assimilations of LAI and SM, and a joint assimilation of LAI + SM were tested and validated using field measurement observation in the city of Hengshui, China, during the 2016–2017 winter wheat growing season. The results indicated that the accuracy of wheat yield simulated by WOFOST was significantly improved after joint assimilation at the field scale. Compared to the open-loop estimation, the yield root mean square error (RMSE) with field observations was decreased by 69 kg/ha for the LAI assimilation, 39 kg/ha for the SM assimilation and 167 kg/ha for the joint LAI + SM assimilation. Yield coefficients of determination (R^2^) of 0.41, 0.65, 0.50, and 0.76 and mean relative errors (MRE) of 4.87%, 4.32%, 4.45% and 3.17% were obtained for open-loop, LAI assimilation alone, SM assimilation alone and joint LAI + SM assimilation, respectively. The results suggest that LAI was the first-choice variable for crop data assimilation over SM, and when both LAI and SM satellite data are available, the joint data assimilation has a better performance because LAI and SM have interacting effects. Hence, joint assimilation of LAI and SM from Sentinel-1 and Sentinel-2 at a 20 m resolution into the WOFOST provides a robust method to improve crop yield estimations. However, there is still bias between the key soil moisture in the root zone and the Sentinel-1 C band retrieved SM, especially when the vegetation cover is high. By active and passive microwave data fusion, it may be possible to offer a higher accuracy SM for crop yield prediction.

## 1. Introduction

Estimation of crop growth parameters at a fine scale over a large area is critical from a food security perspective, because it provides information crucial to many agronomical applications [1,2,3]. Differing from traditional yield statistical estimation methods, crop growth models such as the Decision Support System for Agrotechnology Transfer (DSSAT) [4], Word Food Studies (WOFOST) [5], and Agricultural Production System Simulator (APSIM) [6] can dynamically describe fundamental processes such as photosynthesis, respiration, biomass partitioning and soil dynamics. However, these crop growth models are driven by a large amount of input parameters (weather, model parameters and agromanagement), which are not always available during the growing season and at regional scale. Remote sensing data can play an important role for reducing uncertainties in crop simulation results at regional scale [7,8,9]. Therefore, crop growth states and final yield cannot be estimated on the regional scale using only crop models; instead, ancillary tools must be used. One of the most promising techniques is the joint use of a crop model with field observation or remote sensing data to estimate crop production. Data assimilation (DA) technology is a method that optimally combines crop models with remote sensing data. The combination of crop models, remote sensing observation and DA provides an effective way to improve simulated crop yield at the regional scale [10,11,12,13].

In the past decades, many types of DA have been developed for crop yield estimations. These works include two main assimilation strategies that are used to reduce the discrepancy between remotely sensed data and simulations. These strategies work either by re-initializing and re-parameterizing using optimization algorithms [14,15,16] or by adjusting the model state variables directly through sequential data assimilation techniques [17,18,19]. When the crop model DA was used to estimate crop yield, the results were always subjected to errors because of the uncertainties in the model’s structure, input data, and parameters. The uncertainties of model structure and input data were not been considered in the model parameter optimization strategy. Sequential DA is a robust method of combining remotely sensed data and models to minimize the uncertainty of a given modeled state and improve the crop yield estimations, as this assimilation enhances the use of error information between the model and observations. Many studies have used the DA for crop growth monitoring and yield estimation, such as the ensemble Kalman filter (EnKF) used in the hydrologic model for agricultural drought monitoring [20], the particle filter (PF) used in the DSSAT-CERES (crop environment resource synthesis) to improve wheat yield estimation accuracy [21], and the four-dimensional variational data assimilation (4DVar) used in the WOFOST model to improve regional crop yield estimates [15]. Among these assimilation algorithms, the EnKF was the most frequently used in crop model DA, because of its ease of implementation, computational efficiency and optimum performance [22,23,24,25].

Most publications about crop DA applications address the assimilation of a single state variable such as leaf area index (LAI) [14,17], soil moisture (SM) [26,27], and biomass [28]. However, many state variables interact in crop growth models and affect the final yield [29]. In recent years, with greater availability of observations, multivariate DA has received much attention in crop model simulations. Pauwels et al. [30] used the hydrology model TOPLATS coupled with WOFOST to assimilate these data with the EnKF. Their study shows that soil moisture data helped improve LAI estimates during the growth season, but the LAI hardly improved the soil moisture characterization, whereas the best results were obtained when the LAI and SM were jointly assimilated. Huang et al. [31] jointly assimilated the MODIS LAI and ET products into the (Soil, Water, Atmosphere and Plant) SWAP model with an optimization algorithm for winter wheat yield estimation. They found that the joint assimilation of the MODIS LAI and ET data performed better than just assimilating the MODIS LAI data or ET (Evapotranspiration) data at the county level. Li et al. [32] jointly used the LAI and CAN (canopy nitrogen accumulation) to calibrate the parameters and initial states of the DSSAT-CERES model to improve the grain yield and protein simulation of winter wheat. Ines et al. [12] analyzed the assimilation of remotely sensed SM from AMSR-E and LAI from the MODIS in DSSAT-CERES for maize yield prediction. The results suggest that the yield prediction accuracy improved more when both SM and the LAI were assimilated. However, the accuracy of the DA results did not improve on a regional scale, because the AMSR-E soil moisture product (0–2 cm depth) at 25 km is biased for fully covered crop land.

The recent deployment of (European Space Agency) ESA’s Sentinel satellites has established a new paradigm for agricultural applications. The optical satellite Sentinel-2 (S2) provides well-suited spectral and temporal data for LAI retrievals at a high resolution [33,34]. The Sentinel-1 (S1) satellite with a C band SAR (synthetic aperture radar) sensor also has demonstrated its ability to retrieve SM over vegetation-covered surfaces [35,36]. The S1 and S2 mission offers approximately five days of regular temporal coverage data with a spatial resolution of 10 m when both A and B satellites were considered. Hence, the S1 and S2 data have the potential to further improve DA schemes for crop growth modeling at the regional level.

The objective of this study is to implement a joint LAI and SM assimilation scheme for S1 and S2 data in the WOFOST model to simulate the winter wheat yield at the field scale and to evaluate its performance at the field and regional scales. The lookup table method was used to retrieve the LAI with S2 data (S2-LAI), and the change detection approach was used to retrieve SM with S1 and S2 data (S1-SM). The retrieval error was determined with field measurements. Four experiments for winter wheat yield estimation were carried out in the city of Hengshui, including open-loop, DA with the S2 LAI alone, DA with S1 SM alone and DA with both the S2 LAI and S1 SM. The sequential data assimilation algorithm EnKF was implemented in this study to assimilate remotely sensed data in the WOFOST. The joint assimilation wheat yield estimation at the field scale and the spatial heterogeneity at the county level were evaluated. Thus, it is highly beneficial to improve the regional winter wheat yield estimation by using multivariable data assimilation with high spatiotemporal satellite data.

## 2. Materials and Methods

### 2.1. Study Area

**Figure 1:**
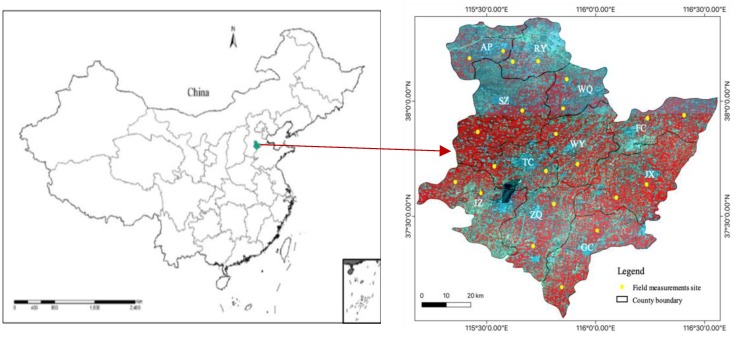
The location of the study area and field observation sites (yellow dots) of winter wheat in 2017.

The study area, the city of Hengshui (37.05°–38.38°N, 115.28°–116.62°E), is located in the southeastern Hebei Province, which is a typical region in the North China Plain. This area covers approximately 8815 km^2^. The area consists of 11 counties, including Taocheng (TC), Shenzhou (SZ), Jizhou (JZ), Gucheng (GC), Zaoqiang (ZQ), Raoyang (RY), Wuyi (WY), Fucheng (FC), Jixian (JX), Wuqiang (WQ), and Anping (AP). The altitude ranges from 12 m to 30 m and is slightly higher in the northwest and lower in the southwest. This area has a semi-humid climate with an annual mean temperature of 12–13 °C and an annual average precipitation of 500–900 mm. The winter is dry and requires irrigation, summers are wet. Most of this area contains agricultural lands with double cropping, including winter wheat, maize and cotton. The soil types in this area are mainly fluvo-aquic soil, wet fluvo-aquic soil and sandy fluvo-aquic soil. The main water source for farmland irrigation in this area is extracted from groundwater. In recent years, comprehensive agricultural experiments have been successfully performed in this region [32,37,38]. In this area, the growing cycle of winter wheat occurs from early October to mid-June of the following year. For winter wheat, seeding occurs in early October, the green-up period begins in early March, the jointing stage lasts from late March to early April, the heading-flowering stage occurs from late April to early May, the milking stage begins in mid-May to late May, and the harvest is usually in mid-June. Figure 1 shows the study area and the distribution of field measurement sites.

### 2.2. Datasets and Processing

#### 2.2.1. Field Observation Data

To validate the model simulation in our study area, field experiments were performed during the 2017 winter wheat growing season in each county of Hengshui city. In the field experiments, we obtained the important biological parameters of the winter wheat, including the LAI, SM, chlorophyll content of wheat leaves, and above ground dry biomass. To validate the remotely sensed data and wheat yield assimilation results at the regional scale, 22 sample plots of homogenous wheat areas were selected from March to June 2017. Each county has two sample plots (Figure 1), and their positions in the field were located using a Trimble GeoXT3000 GPS from Trimble Navigation, Ltd. The wheat LAI of different phenological stages (green-up, jointing, heading, and milking) were measured by the LAI-2000 plant canopy analyzer from LI-COR Inc., and the surface SM was measured at 10 cm depth by FieldScout TDR300 from Spectrum Technologies Inc. The winter wheat yields were measured at 22 field plots during the wheat maturity stage. The winter wheat field measurements in 2014 from Li et al. [21] were also collected to calibrate the WOFOST model.

#### 2.2.2. Weather, Soil and Crop Data

The fundamental data inputs for crop growth models include weather data, soil data and crop management data. The weather data of 11 meteorological stations (one station in each county) for the period 2016–2017 were acquired from the National Meteorological Information Center, China Meteorological Administration, including the daily maximum and minimum air temperature, rainfall, wind speed, and sunshine duration. Since solar radiation is needed by the WOFOST, the Angstrom formula from the FAO-56 guide was used to convert sunshine hours into solar radiation [39]. The soil data used for this study were obtained from the 1:1,000,000 China Soil Database. The soil’s physical and chemical properties can be obtained from the soil profiles. Detailed crop management information was also surveyed during the field experiments, including the planting dates, planting depth and spacing, planting density, irrigation dates and volumes, fertilization dates and volumes, and other data.

#### 2.2.3. Remote Sensing Data and Retrieval

The optical satellite images from Sentinel-2 and the SAR satellite images from Sentinel-1 during the growing season were employed in this study. Sentinel-2 has two satellites, the Sentinel-2A (S2A) satellite was launched in June 2015, which was followed by Sentinel-2B (S2B) in March 2017. The S2 mission is designed to provide full and systematic coverage of the Earth’s land surface, which offers a wide-swath, high-resolution and multispectral images [40]. The S2 data corresponds to images recorded in 13 spectral bands, with three spatial resolutions of 10 m, 20 m and 60 m dependent on the particular spectral band. The SNAP (sentinel application platform) toolbox Sen2Cor was used for S2 data atmospheric correction [41]. The SAR images were obtained from the Sentinel-1 mission, which also launched two satellites, Sentinel-1A (S1A) in April 2014 and Sentinel-1B (S1B) in April 2016. Both the S1A and S1B operate in the C bands, with VV and VH polarizations. All the Sentinel-1 data were pre-processed using the Sentinel-1 Toolbox, including: radiometric calibration, multilooking, filtering, and terrain correction [36]. In this study, the S1 images used were generated from the Interferometric Wide (IW) swath mode and Level-1 Ground Range Detected (GRD) products.

For the growth of winter wheat, a series of S1A and S2A free cloud images were acquired from February to June 2017 (Table 1). A lookup table (LUT) method based on the PROSAIL model was used to extract regional winter wheat LAI from S2A images [34]. The retrieved LAI data had a spatial resolution of 20 m. Band 4 (Red) and band 8 (NIR) were used to calculate the NDVI and resample to 20 m. For the high vegetation cover, previous studies have shown that VH data were not suitable for the estimation of SM due to high sensitivity to volume scattering [42,43]. Recently, some studies have shown that there is a large potential for mapping SM at a high spatial resolution in agricultural areas through the synergic use of S1 and S2 images [36,44,45]. Therefore, the S1A VV data and S2A NDVI data were selected to retrieve SM. The inversion approach was a change detection method, which is based on the difference in backscattered signals observed on two consecutive days of S1A data. This method assumes that the change in vegetation is very small and that the difference in backscattered signals mainly depends on the change in SM [45]. Therefore, the difference in the backscattered signal is a function of NDVI (retrieved from S2A data). The retrieved SM data also have a spatial resolution of 20 m.

The retrieved data were compared with field measurements to obtain the observation errors, i.e., coefficient of determination (R^2^), mean relative error (MRE) and root mean square error (RMSE) between the retrieved and measured data.

Before LAI and SM retrieval, the time series data of S2A NDVI were used to extract the spatial distribution map of winter wheat in 2017. The winter wheat area map with 20 m spatial resolution been used as a mask to retrieve crop-specific observed data that was used to perform the data assimilation with the WOFOST at the regional scale.

## 3. Method

### 3.1. WOFOST Model and Calibration

In this study, we used the WOFOST model to simulate growth of winter wheat. The WOFOST is a mechanistic model that can properly describe the dynamic growing process of crops, such as phenological development, photosynthesis, assimilation and respiration, biomass increase and partitioning, and grain yield [18]. The WOFOST was built by Wageningen University in the Netherlands. The WOFOST model describes plant growth using the phenological development of the crop as the growth controlling process and uses light interception and CO_2_ assimilation as the growth driving processes. The crop development stages (DVS) are expressed using a dimensionless variable, starting at zero representing emergence, one representing anthesis, and two representing maturity. There are three production levels implemented in the WOFOST model: the potential level, water limited level and nutrition limited level. The output variables include daily results such as the LAI, SM, above ground dry biomass, and grain yield, and the summary results such as the anthesis date, maturity date, and maximum LAI. Since the WOFOST model was rewritten in pure python and embedded within the Python Crop Simulation Environment (PCSE) [5,18], we implemented winter wheat DA with the WOFOST based on this environment. The PCSE is a python package for building crop simulation models. The PCSE provides the environment to implement crop simulation models, tools for reading ancillary data and components for simulating biophysical processes. Implementations of the WOFOST model are included in the PCSE, which are easier to understand and more convenient to use.

Different crops were designed with different default crop parameter sets in the WOFOST model. The parameter sensitivity of the WOFOST model was obtained in previous studies[46]. During crop growth, the LAI is an important WOFOST model state, which are the functions of a few variables, including the specific leaf area (SLATB), dry matter weight of leaves (LV), physiological age of leaves (LVAGE) and the class number of living leaves (IVOLD). Leaf growth and senesce together determine the status of the LAI. SPAN and TBASE are two key parameters determining physiological aging. The SPAN parameter represents the maximum leaf age at 35 °C, and the TBASE parameter is defined as the lower threshold temperature for physiological aging. The SM is also an important parameter for crops because it can affect the crop’s respiration and transpiration, which are very important processes in crops’ vital movement. The interaction between the SM and crop growth is critical for drought-sensitive crops when soil moisture availability limits growth. The SM, especially surface soil moisture, and LAI, are two of the most common observations.

In this study, the water limited mode of the WOFOST model was used to simulate the daily LAI, SM, and grain yield at the maturity stage. Field measurements obtained during 2014 in our study area were used for WOFOST model parameterization and calibration [32,37].

### 3.2. Ensemble Kalman Filter

In a sequential data assimilation approach, the model parameters and state variables can be updated when new observations are available. As one of the sequential data assimilation method, the EnKF is commonly used for crop yield improving simulation results of highly nonlinear crop growth models [12,18,22]. The theoretical framework of the EnKF algorithm was proposed by [47], which is essentially a Monte Carlo implementation of the Kalman filter. By assuming Gaussian distributions of model error and observation uncertainties, the EnKF generates an ensemble of realizations model states to approximate the probability distributions of the priori states. The process of the EnKF is divided into two steps: forecast (prior to update) and analysis (updated).

In the forecast step, each ensemble member of the state variable can be written as:

(1) $$X_{i,t}^{f}=M(X_{i,t-1}^{a},\alpha_{t},\beta_{t})+\omega_{i}\sim N(0,Q)$$

where, M(·) is the model operator of the WOFOST model in this case; the superscripts f and a represent the forecast and analyzed estimates, respectively; α and β are the weather forcing data and model parameters. The model error $\omega_{i}$, which represents all the model uncertainties, is assumed as a Gaussian distribute of zero mean and the covariance matrix Q. With the *N*_e_ ensemble members, the ensemble mean and covariance matrix of forecast state variable are:

(2) $${\bar{X}}_{t}^{f}=\frac{1}{N_{e}}\sum_{i=1}^{N_{e}}X_{i,t}^{f}$$

(3) $$P_{t}^{f}=\frac{1}{N_{e}-1}\sum_{i=1}^{N_{e}}(X_{i,t}^{f}-{\bar{X}}_{i,t}^{f}){\left(X_{i,t}^{f}-{\bar{X}}_{i,t}^{f}\right)}^{T}$$

When an observation is available, the observation data is assimilated into the model to update the state variable. A perturbation is added into the actual observation to represent the observation error. The error is conforming to Gaussian distribution with zero mean and the covariance matrix R. The generated observation ensemble equation as following:

(4) $$Y_{i,t}^{\mathrm{obs}}\;=\;y_{t}^{\mathrm{obs}}\;+\;v_{i,t}\sim N(0,R)$$

In the analysis step, the prior estimation and the difference between the observation data and the prior estimation of these data in forecast step were used to update the state variable in each ensemble member. Then, each ensemble member of forecast state variable is updated with the following equation:

(5) $$X_{i,t}^{a}=X_{i,t}^{f}+K_{t}[Y_{t}^{\mathrm{obs}}-H(X_{i,t}^{f})]$$

where, H(·) is observation operator used to convert the model state to observation space. In this study, as the model state variable LAI and SM is observed directly, the observation operator H is an identity matrix. $K_{t}$ is the Kalman gain at time t, which is calculated as:

(6) $$K_{t}=P_{t}^{f}H^{T}{\left(HP_{t}^{f}H^{T}+R\right)}^{-1}$$

Finally, the state variable of each ensemble member updated by considering the available observation data and the given model at time t. Then, the ensemble mean ${\bar{X}}_{t}^{a}$ and covariance matrix $P_{t}^{a}$ of analysis state variable are calculated same as Equations (2) and (3). The updated ensemble is then integrated forward until the next observation variable, and the process is repeated with the above method.

### 3.3. Data Assimilation Schemes

In this study, the LAI and SM were taken as the assimilation state variables of EnKF DA, and these two variables obtained directly from observations. Based on the retrieval validation with the field measurement, 10% perturbation of retrieved LAI and 35% perturbation of retrieved SM were used to calculate the observational variance for the EnKF assimilation. In this study, the uncertainties in the model structure and input data are ignored, and only the uncertainty in the initial variables and model parameters were considered. During the EnKF implementation, the initial variables and parameters were perturbed according to the prior information to generate ensemble members [46,48]. An ensemble size of 50 is chosen in this study. The initial variable TDWI (initial total crop dry weight) and WAV (initial soil water content), the sensitivity parameters for LAI: RGRLAI (maximum relative increase in LAI) and SPAN, and the sensitivity parameters for SM: SMFCF (field capacity of soil) and SM0 (soil saturated water content) were perturbed with a 10% uncertainty. The parameters’ initial values were obtained from the calibration through field measurements in 2014 and assumed as Gaussian distributions. The other parameters’ values were obtained from the calibration and previous studies [15,17,49]. At every LAI or SM observation time, the EnKF updates the states and then propagates the samples through time using the forward equations.

To introduce the jointly assimilated Sentinel retrieved LAI and SM into the WOFOST model and evaluate its performance, a field scale experiment for winter wheat estimation was performed in this study. The WOFOST model began running on the sowing dates based on the field measurements. There are three DA schemes for wheat yield simulation: assimilating the LAI alone, assimilating the SM alone, and assimilating the LAI and SM jointly. All the assimilation schemes were performed with the same simulation conditions, including model inputs, initial conditions, and parameters. The assimilation results were compared with the open-loop simulation. At the field scale, the pixels of S2 retrieved the LAI and S1 retrieved the SM corresponding to each field plot were extracted as the observations. The performance of the WOFOST DA for wheat yield was evaluated using field wheat yield measurements. The yield simulation results of the three DA schemes and open-loop runs were compared using statistics such as the R^2^, RE, and RMSE. At the regional scale, consistent with the interpolated weather data, the 1 km scale of the retrieved LAI and SM were used in the assimilation procedure. In the 1 km grid cells, the model ran only when winter wheat pixels were greater than 40%, and the average of LAI and SM was calculated. The winter wheat yields at the county level were calculated to analyze weather the joint assimilation can improve the spatial heterogeneity simulation ability of the WOFOST model.

## 4. Results

### 4.1. Sentinel Retrieved LAI and SM Validation with Ground Measurements

**Figure 2:**
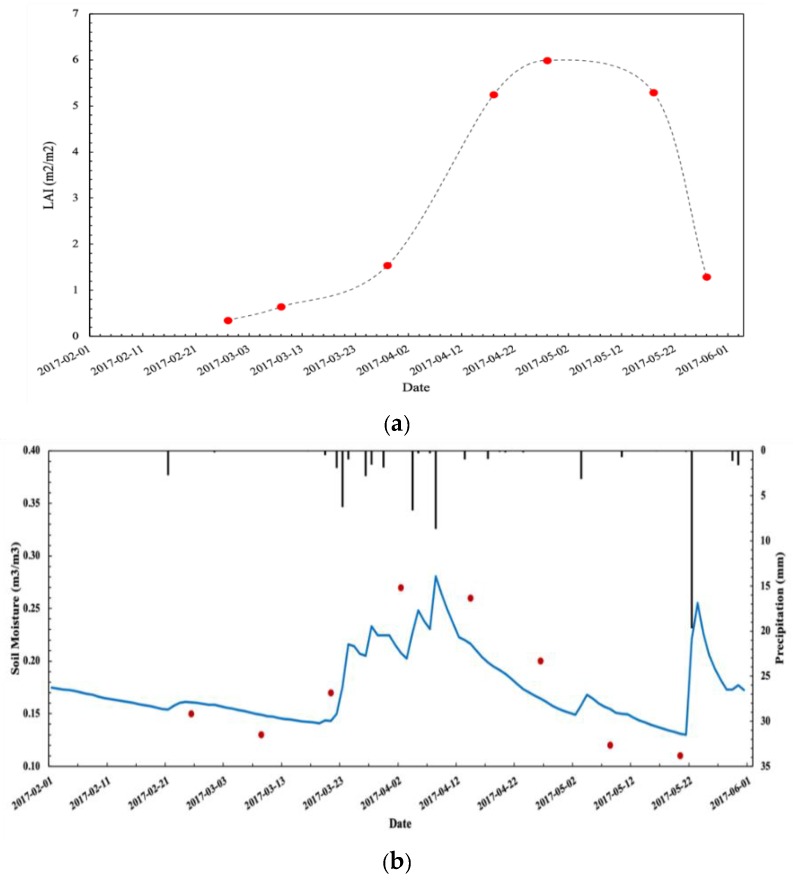
Temporal variations in retrieved leaf area index (LAI) (**a**) from Sentinel-2 and retrieved soil moisture (SM) (**b**) from Sentinel-1 at the Raoyang site. Temporal variation in S2 retrieved LAI (red dot) at the Raoyang site. Temporal variation in S1 retrieved SM (red dot) and in situ measurements at the Raoyang site.

**Figure 3:**
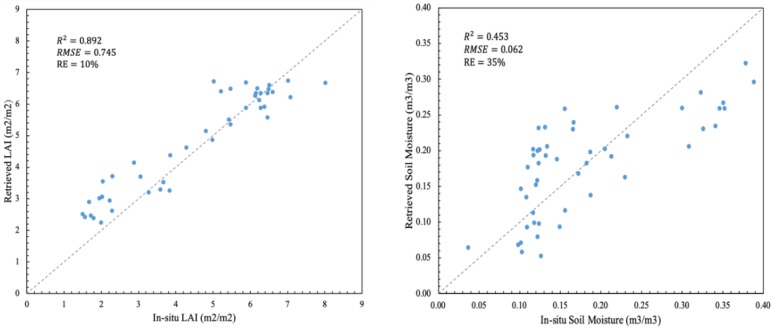
Scatterplot of retrieved LAI versus in situ LAI values (**left**) and retrieved SM versus in situ SM values (**right**) evaluated in the study area.

In this study, between winter wheat green-up and maturity in 2017, seven LAI images were obtained using the lookup table method and eight SM images were obtained using the change detection method. Using the ground measurements recorded in the 22 sample plots from March to June 2017, we compared the S2 retrieved LAI with measured LAI and the S1 retrieved SM, with surface moisture measurements obtained at a depth of 10 cm in the field. The series key phenological stages of wheat, such as green-up, jointing, heading, anthesis and maturity, have been obtained from the time series data of S1 and S2. Figure 2 shows the temporal variation in the LAI and SM retrieved from satellite data during the wheat growth period at the RY field observation site. The LAI values retrieved from the S2 data as a function of time well describe the process of wheat leaf growth and senescence. The SM values retrieved from the S1 data are also well correlated with the observed moisture and precipitation events, with the soil moisture increasing after each significant rainfall event. Figure 3 compares the retrieved LAI and SM values with the ground measurements. The results showed that the retrieved LAI has a high accuracy, with an R^2^ of 0.892, an MRE of 10% and an RMSE of 0.745. For the retrieved SM, the accuracy of the inversion is not as good as the retrieved LAI, with an R^2^ of 0.453, an MRE of 35% and an RMSE of 0.062. The validation results showed that the retrieved LAI from S2 accurately represented the LAI values during the wheat growth periods, and although the precision of retrieved SM from S1 is relatively low, the temporal changes in LAI and SM are acceptable and could be employed in the crop model DA.

**Figure 4:**
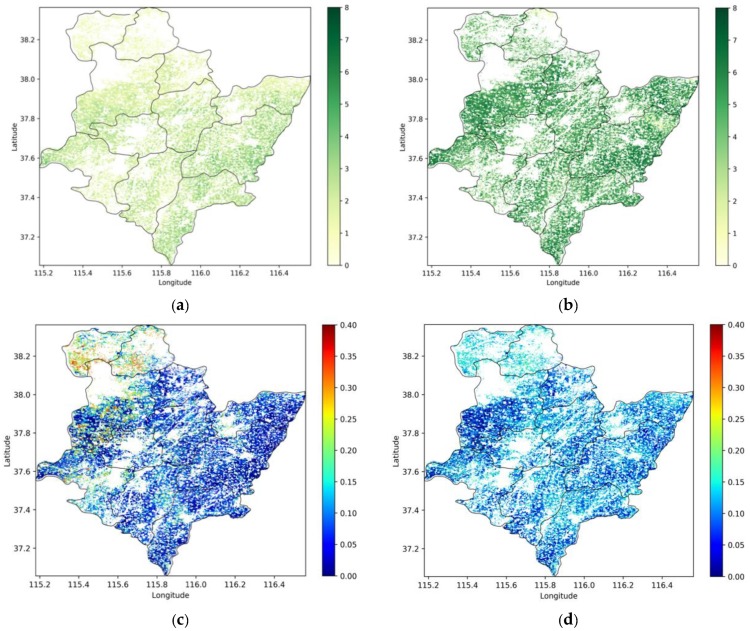
Retrieved LAI and SM maps obtained at two key phenological stages (green-up: (**a**, **c**); and jointing: (**b**, **d**)) of winter wheat area in the city of Hengshui. (**a**) Retrieved LAI 29 March 2017. (**b**) Retrieved LAI 28 April 2017. (**c**) Retrieved SM 2 April 2017. (**d**) Retrieved SM 8 May 2017.

Figure 4 shows the spatial distribution of winter wheat fields LAI and surface SM at two key phenological stages: the green-up stage and jointing stage. From the LAI maps of the study area, the LAI values in the south are slightly higher than those in the north. Thus, the southeast may have an earlier phenological stage than the north. The spatial difference in surface soil moisture is also shown in the SM maps. We found that the SM values are high in the green-up stage, which is also a wheat irrigation stage. Given the spatial heterogeneity in the LAI and, in particular, the SM maps, it is clear that the data assimilation can improve the simulation of wheat yields at regional scale [50].

### 4.2. Impact of Assimilating LAI and SM into WOFOST

**Figure 5:**
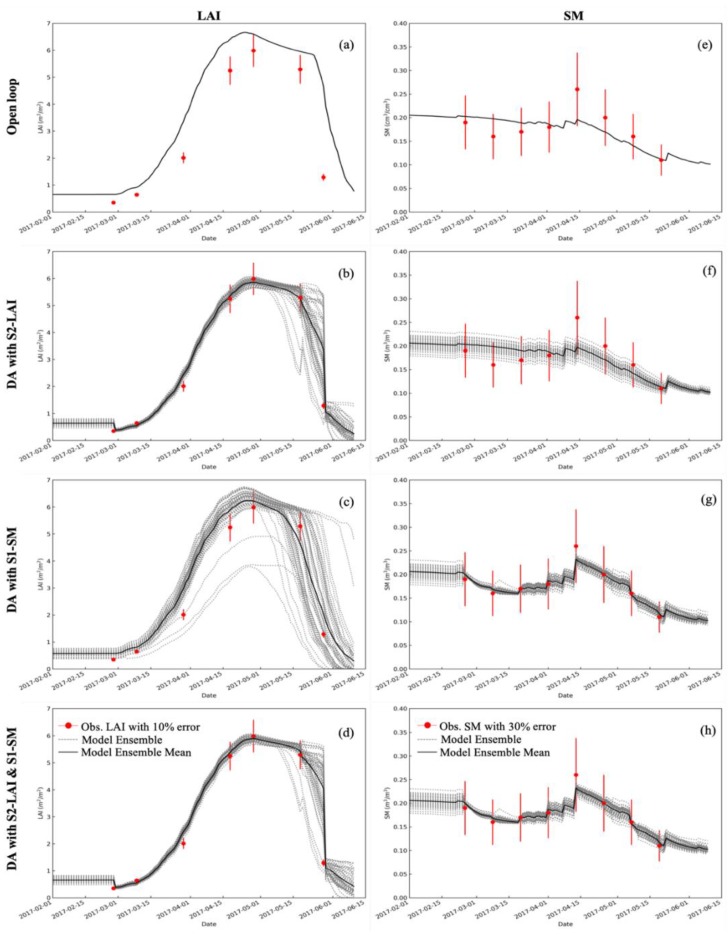
The LAI and SM simulations of the Word Food Studies (WOFOST) with open-loop (**a**,**e**), assimilation of S2-LAI (**b**,**f**) and S1-SM (**c**,**g**) respectively, and joint assimilation of S2-LAI and S1-SM (**d,h**) at the Raoyang site in 2017.

The assimilation of LAI data into the WOFOST has little impact on the water balance, and the assimilation of SM data has an effect on canopy growth. Figure 5 shows the evolution of the simulated LAI and SM at the RY site during the 2017 wheat growth, with open-loop (Figure 5a,e), assimilation of S2-LAI alone (Figure 5b,f), assimilation of S1-SM alone (Figure 5c,g), and joint assimilation of S1-SM + S2-LAI (Figure 5d,h). The results of the model ensemble and observations with errors are also presented in Figure 5. Assimilating S2-LAI or S1-SM independently significantly improved the simulation of LAI or the root SM in WOFOST (Figure 5b,g) compared to no DA. For LAI simulation, improvements in surface SM estimations contribute to better estimations of LAI (Figure 5c). Ines et al. [12] observed that assimilating remotely sensed SM into a crop model can improve the simulation under drought conditions. Contrarily to LAI results, the assimilation of LAI alone did not significantly improve the simulated SM (Figure 5f). In Figure 5d,h, the impact of the joint S2-LAI and S1-SM analysis are shown for the LAI and SM predictions. The predicted values are quite close to the observations, improving the performance of the wheat growth simulation.

### 4.3. Impact of Assimilating Sentinel Data on Simulated Yield at Field Scale

To explore the performance of the joint LAI and SM assimilation for crop yield estimation, we assimilated the S1-SM and S2-LAI values into the WOFOST model based on the data from the Hengshui measurements at the field scale. In this experiment, a series of S2 retrieved LAI and S1 retrieved SM in 22 field plots were assimilated into the WOFOST model. Three assimilation schemes (DA with LAI, DA with SM, and DA with LAI+SM) were applied to the crop model assimilation at the field scale. Seven S2-LAI values and eight S1-SM values were assimilated at each field plot. The same model inputs, and initial conditions were used in the model runs. The simulated yields of open-loop run and assimilation schemes were compared with the field measured yields.

**Figure 6:**
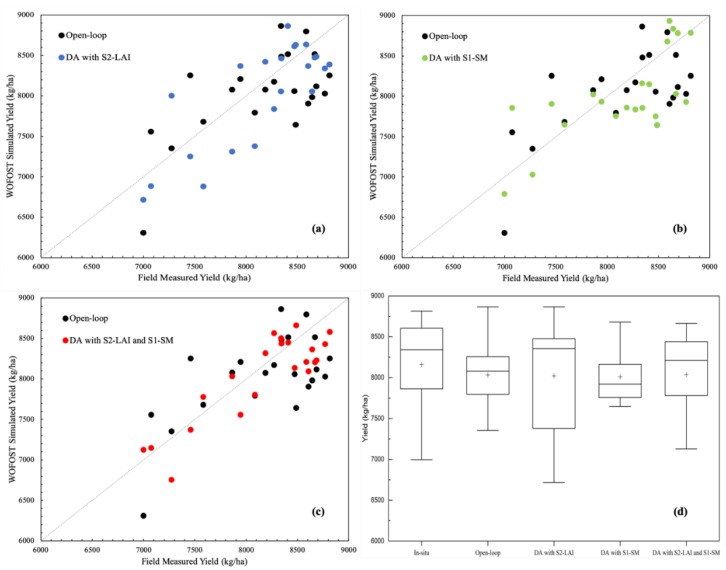
Comparisons of winter wheat yield estimated by the WOFOST model with open-loop and different DA strategies over the field scale in Hengshui in 2017.

The winter wheat yields of 2017 at the field sites were simulated using the calibrated WOFOST model. The accuracy of estimated yields with open-loop and DA were analyzed based on the R^2^, MRE and RMSE, which are listed in Table 2 and plotted in Figure 6. The open-loop was able to capture the difference in yields between different field sites. The uncertainties in the input data, model structure and parameters are the main error sources of the open-loop. Compared to the open-loop, the yield RMSE with field observation was decreased by 69 kg/ha for the LAI assimilation, 39 kg/ha for the SM assimilation and 167 kg/ha for the joint LAI + SM assimilation. The yield coefficients of determination (R^2^) of 0.65, 0.50, and 0.76 and an MRE of 4.32%, 4.45% and 3.17% were obtained for the LAI, SM and joint LAI+SM assimilation, respectively, compared to the open-loop with an R^2^ of 0.41, and MRE of 4.87%. The yield boxplot (Figure 6d) shows that the simulated yield of joint S2-LAI and S1-SM assimilation and in situ yield have the best consistency.

### 4.4. Assimilation of Sentinel Data for Winter Wheat Yield Mapping at the Region Scale

**Figure 7:**
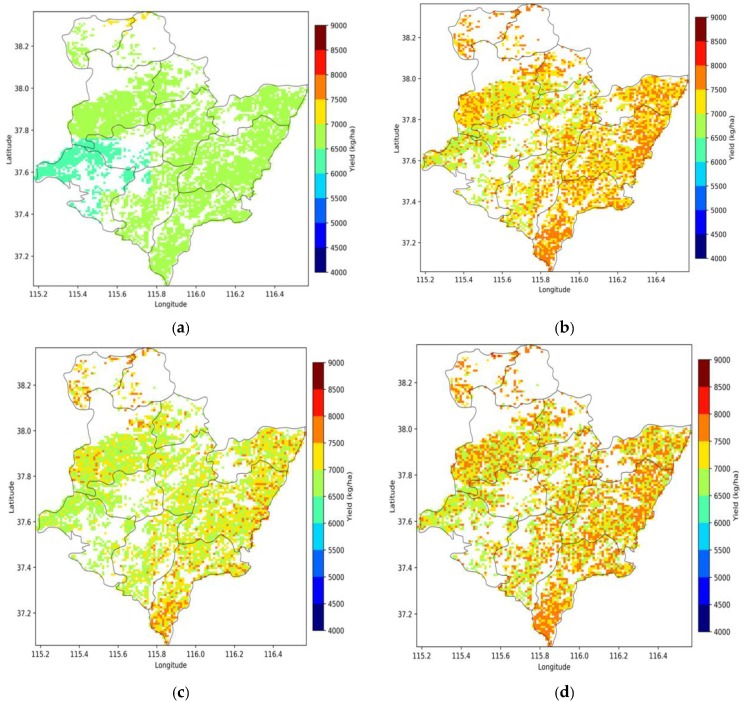
The winter wheat yield simulation with different data assimilation schemes ((**a)** open-loop; (**b)** DA with LAI; (**c)** DA with SM; and (**d)** DA with SM+LAI). (**a**) Open-loop, (**b**) DA with LAI, (**c**) DA with SM, (**d**)DA with LAI+SM.

At the regional scale, considering the computing cost and weather data scale, the WOFOST model was applied at 1 km grid cells when the fractional coverage of wheat within that pixels was greater than 40%. The remotely sensed LAI and SM values in the compliant grid cells were averaged and then assimilated into the WOFOST to map the spatial distribution of winter wheat yield. We compared the yield map of the open-loop runs with the maps simulated by three assimilation schemes: DA with LAI alone, DA with SM alone, and DA with LAI+SM (Figure 7). The accuracy of the wheat yield estimation was evaluated at the pixel scale using the in situ yield measurements. The simulated yields of the open-loop were much lower than the field observation yields, with an R^2^ of 0.13, and an RMSE of 1873 kg/ha. The accuracy of assimilation with LAI alone is higher than the assimilation with SM alone. The joint assimilation of LAI and SM achieved the highest accuracy, with an R^2^ of 0.53 and the lowest RMSE of 709 kg/ha. However, the yields of the field measurements were usually higher than the official statistical yields [21], so the verification at the county level is required when the statistical data available. Overall, the yield maps showed that the yield per unit area in the east was higher than that in the west in this study area. The yield map obtained without DA did not well reflect the spatial variability. The spatial variability of the wheat yield in the assimilation using both LAI and SM was good at the regional scale due to the higher spatial detail of the crop growth condition information. It is clearly visible in the maps that all DA approaches add spatial variability in the simulated wheat yield that is not present in the open-loop simulation.

## 5. Discussion

The optical data from Sentinel-2 image and SAR data from Sentinel-1 image were used to estimate the LAI and surface SM of winter wheat fields. The retrieved LAI based on the LUT method has high accuracy with an MRE of 10%. The retrieved SM accuracy is a relatively low, with an MRE of 35%, due to the limitation of the C band under high vegetation density. However, as the spatiotemporal resolutions of the S1 and S2 data are high, the dynamic change in the surface soil moisture can be obtained from the retrieved SM time series data. These results demonstrate the potential of S1 and S2 data for obtaining information on wheat LAI and surface crop growth conditions. In this study, the retrieved LAI and SM time series from the S1A and S2A data were assimilated into the WOFOST model using the EnKF algorithm. The winter wheat yield simulation with open-loop, assimilating S2-LAI alone, assimilating SM alone, and joint assimilation of S2-LAI and S1-SM was implemented at the field scale. In this study, the retrieved time series data included some key stages of wheat growth, such as the jointing stage which is the most water sensitive period, and the heading stage, which is the most sensitive stage for wheat yield [11,31,32]. The joint assimilation of S2-LAI and S1-SM has superior performance in estimating crop yield compared to assimilating S2-LAI and S1-SM data independently at the field scale. Assimilation of the S2-LAI was more useful than assimilation of S1-SM, which is consistent with the conclusions of Ines et al. [12], who indicated that SM assimilation may not be necessary under very wet conditions.

Before the joint assimilation implementation of remotely sensed LAI and SM into the WOFOST model, the assimilation of LAI alone and of SM alone was attempted. The assimilation of the LAI alone did not improve the simulated SM under normal soil water conditions. However, that the assimilation of SM alone improved the simulated LAI is obvious, as crop canopy growth was improved under the water-limited mode of the WOFOST model. Previous studies have shown that the SM was more sensitive to yield than the LAI at the milking stage, as the wheat leaves turned yellow during this stage, and the assimilation of LAI was more important than SM assimilation [11]. Therefore, when jointly assimilating the LAI and SM into crop models, the crop growth stages should also be considered to determine the best combination of the state vectors.

At the regional scale, the open-loop and DA of the WOFOST model were implemented for all 1 km grid cells with at least 40% fractional coverage of wheat pixels. The DA results at the regional scales showed significant improvement in wheat yield simulation accuracy in cases of using both the S2-LAI and S1-SM in the assimilation process. The results confirmed that assimilating the LAI has a dominant influence in terms of yield improvement. Assimilation of the S1-SM alone slightly improved the wheat yield map, as the retrieved SM only can reflect the surface soil moisture and the high spatial heterogeneity of soil moisture. By evaluating the accuracy of wheat yield estimation at the pixel scale, the yield correction improved more with an R^2^ of 0.53, and the prediction errors RMSE was also reduced by 696 kg/ha when both S2-LAI and S1-SM were assimilated. In this paper, the field yield measurements were used to evaluate the simulated yield at 1 km grid. However, the performance of the LAI and SM joint assimilation into the WOFOST model still needs to be analyzed at the county level.

Integrating S1 and S2 retrieved the LAI and SM data with the WOFOST model presents great potential for crop grain yield estimations. As a limitation of S1 data for root soil moisture monitoring, the high-resolution SMAP/Sentinel-1 soil moisture product may be used to further improve the crop yield estimation at the regional scale. Notably, for multivariate DA, the timing and frequency of assimilation data are related to the crop growth stages [11,12,31].

## 6. Conclusions

The joint assimilation of Sentinel-1 and Sentinel-2 retrieved LAI and SM in order to update model state variables in WOFOST using the EnKF algorithm was tested in this study. Four strategies (open-loop, LAI assimilation alone, SM assimilation alone and joint LAI-SM assimilation) were analyzed and compared with field observed data. The temporal evolutions of the LAI and SM were significantly improved by assimilation. At the field scale, our results showed that joint assimilation of S2-LAI and S1-SM can lead to accuracy improvements and uncertainty reductions compared to single state variable assimilation. This assimilation scheme of remotely sensed LAI and SM joint assimilation can also aid in wheat yield mapping at the regional scale. However, implementation of the WOFOST model at the regional scale cannot depend solely on remotely sensed DA because estimates may substantially diverge from reality. The weather data and model structure will also contribute to yield simulation uncertainties. In addition to the most easily applied LAI and SM data, evapotranspiration (ET), biomass or canopy nitrogen content were required to improve DA performance. With the emergence of new remote sensing technology, near real-time data with an adequate spatial resolution has become available for agricultural applications. Therefore, we need to pay more attention to acquiring and assimilating multivariate information in the crop model data assimilation.

## Figures and Tables

**Table 1:** Sentinel-1A and Sentinel-2A datasets used in this study.

Sentinel-1A		Sentinel-2A	
Date	Date	Date	Date
25 February 2017	26 April 2017	27 February 2017	18 May 2017
09 March 2017	08 May 2017	09 March 2017	28 May 2017
21 March 2017	20 May 2017	29 March 2017	
02 April 2017		18 April 2017	
14 April 2017		28 April 2017	

**Table 2:** The performance of the different DA strategies for WOFOST simulating of winter wheat yield at field scale (N = 22), in Hengshui in 2017.

Scheme	R^2^	MRE	RMSE (kg/ha)
Open-Loop	0.41	4.87%	473
DA with LAI	0.65	4.32%	404
DA with SM	0.50	4.45%	435
DA with LAI + SM	0.76	3.17%	306
